# Variable Extent of Lineage-Specificity and Developmental Stage-Specificity of Cohesin and CCCTC-Binding Factor Binding Within the Immunoglobulin and T Cell Receptor Loci

**DOI:** 10.3389/fimmu.2018.00425

**Published:** 2018-03-08

**Authors:** Salvatore Loguercio, E. Mauricio Barajas-Mora, Han-Yu Shih, Michael S. Krangel, Ann J. Feeney

**Affiliations:** ^1^Department of Molecular Medicine, The Scripps Research Institute, La Jolla, CA, United States; ^2^Department of Immunology and Microbiology, The Scripps Research Institute, La Jolla, CA, United States; ^3^Department of Immunology, Duke University Medical Center, Durham, NC, United States

**Keywords:** CCCTC-binding factor, cohesin, long-range looping, immunoglobulin loci, T cell receptor loci, 3D chromatin topology

## Abstract

CCCTC-binding factor (CTCF) is largely responsible for the 3D architecture of the genome, in concert with the action of cohesin, through the creation of long-range chromatin loops. Cohesin is hypothesized to be the main driver of these long-range chromatin interactions by the process of loop extrusion. Here, we performed ChIP-seq for CTCF and cohesin in two stages each of T and B cell differentiation and examined the binding pattern in all six antigen receptor (AgR) loci in these lymphocyte progenitors and in mature T and B cells, ES cells, and fibroblasts. The four large AgR loci have many bound CTCF sites, most of which are only occupied in lymphocytes, while only the CTCF sites at the end of each locus near the enhancers or J genes tend to be bound in non-lymphoid cells also. However, despite the generalized lymphocyte restriction of CTCF binding in AgR loci, the Igκ locus is the only locus that also shows significant lineage-specificity (T vs. B cells) and developmental stage-specificity (pre-B vs. pro-B) in CTCF binding. We show that cohesin binding shows greater lineage- and stage-specificity than CTCF at most AgR loci, providing more specificity to the loops. We also show that the culture of pro-B cells in IL7, a common practice to expand the number of cells before ChIP-seq, results in a CTCF-binding pattern resembling pre-B cells, as well as other epigenetic and transcriptional characteristics of pre-B cells. Analysis of the orientation of the CTCF sites show that all sites within the large V portions of the Igh and TCRβ loci have the same orientation. This suggests either a lack of requirement for convergent CTCF sites creating loops, or indicates an absence of any loops between CTCF sites within the V region portion of those loci but only loops to the convergent sites at the D-J-enhancer end of each locus. The V region portions of the Igκ and TCRα/δ loci, by contrast, have CTCF sites in both orientations, providing many options for creating CTCF-mediated convergent loops throughout the loci. CTCF/cohesin loops, along with transcription factors, drives contraction of AgR loci to facilitate the creation of a diverse repertoire of antibodies and T cell receptors.

## Introduction

The evolutionarily conserved, ubiquitously expressed zinc finger protein CCCTC-binding factor (CTCF) plays many important roles in gene activation and/or gene repression (1). Many years ago, it was shown to be the main protein associated with insulator function, in that it insulates a transgene from variable expression due to integration position effects, and that it also blocks an enhancer from acting on a promoter when the insulator element is in between them (2, 3). Subsequently, it was appreciated that CTCF exerts essentially all these activities by virtue of its ability to create long-range loops, thus creating domains within which promoters and enhancers can interact (4–7). By contrast, interactions of promoters in one domain and enhancers in another seldom happen. More recently, it has been appreciated that the entire genome is organized into “topologically associating domains” (TAD), which are megabase-sized chromatin domains that are largely conserved across different cell types (8–10). The boundaries of almost all TADs are bound by CTCF, and thus CTCF is now well established as one of the major architectural proteins organizing the entire genome (8, 10–13). Since TADs are generally conserved among various cell types and even conserved across species, it is not surprising that the majority of CTCF sites are invariant across different cell types (8, 13, 14). However there are also CTCF sites that are cell type specific and which correlate with cell type-specific gene expression (14, 15).

Cohesin is often found bound at CTCF sites genome wide (16–18), and recent findings suggest a key role for cohesin in the long-range looping of CTCF to create TADs (19). According to the loop extrusion model, the cohesin complex binds to chromosomes and extrudes the chromatin, forming a loop, until it encounters a boundary element, predominantly a CTCF site (20–23). By extruding the chromatin until 2 CTCF sites in convergent orientation to each other are reached, long-range loops with convergent CTCF sites at the base of the loops are formed. Hence, cohesin plays an essential role in the formation of the CTCF loops at TAD boundaries. These loops are not fixed, and cohesin and CTCF will dissociate with time and then the cohesin complex will bind again, and begin the loop extrusion process again until a new CTCF-mediated loop is formed (20, 23, 24).

Lymphocyte differentiation is an ideal model system to study universal vs. lineage-specific vs. developmental stage-specific binding of CTCF since it is easy to isolate pure populations of the sequential stages of differentiation of the B and T lineage progenitors. The developmental pathway of lymphocytes is well worked out, and the rearrangement of antigen receptor (AgR) genes in T and B cells occurs at precisely defined stage of lymphocyte development (25). The process of V(D)J rearrangement creates a wide diversity of immunoglobulins (Igs) and T cell receptors (TCRs) to enable the immune system to combat a wide variety of pathogens and abnormal cells. Rearrangement at each of the B cell and T cell AgR loci occurs at precise stages of differentiation and only in the appropriate lineage of lymphocytes. T and B cells both arise from a common progenitor cell type (26). The first stage of committed B cell development is the pro-B cell stage during which the Igh locus rearranges. Following successful productive rearrangement of an immunoglobulin heavy chain (Igh) gene, the pro-B cells differentiate into the pre-B cell stage during which the Igκ and the Igλ loci rearrange sequentially. T cell precursors migrate from the bone marrow (BM) to the thymus, and they first undergo rearrangement at the CD4^−^CD8^−^ “double-negative” (DN) thymocyte stage, at which time the TCRβ, TCRγ, and TCRδ rearrangements all take place. If both productive TCRγ and TCRδ rearrangements occur, the cell differentiates into a γδ T cell. However, if a productive TCRβ rearrangement precedes productive rearrangement of both the TCRγ and TCRδ loci, the DN thymocyte progenitor will differentiate into a double-positive (DP) thymocyte, at which stage the TCRα genes undergoes rearrangement and the DP thymocyte will then differentiate into an αβ T cell (27).

In each AgR locus, one of the many V gene segments joins to one of the many D or J gene segments to create an enormous diversity of receptors. However, some AgR loci are very large, up to 3 Mb, and the portion of each AgR locus which contains D and J genes covers only a very small fraction of each locus. Since the many V gene segments are spread so far apart, this raises the question of how V gene segments that are very distant from the J gene segments can manage to find the J genes to rearrange. Several years ago, it was shown by 3D fluorescence *in situ* hybridization (3D-FISH) that the Igh locus has a rosette-like structure made by multiple long-range interactions (28). This structure becomes even more compact at the pro-B cell stage of B cell development, the developmental stage when the Igh locus undergoes V(D)J rearrangement (28–30). This process of locus contraction brings the Vh genes, spread over 2.5 Mb, into closer proximity to the D and J genes to which one Vh will rearrange to create a functional VDJ exon encoding the variable antigen-binding part of the Igh protein. The other AgR loci were also shown to undergo locus contraction at or prior to the developmental stage when they undergo rearrangement (31–34).

We previously hypothesized that a protein such as CTCF, with its ability to make long-range loops, might be responsible for creating the rosette-like structure at the Igh and presumably at other AgR loci, and might also contribute to locus contraction (35). If this were a reasonable hypothesis, then there would need to be many CTCF and cohesin sites within the AgR loci, and if they contributed to locus contraction, CTCF binding might be increased in an AgR locus at the specific stage of B or T cell development at which that AgR locus undergoes rearrangement. In order to see if this were a viable hypothesis, we performed ChIP-chip, and subsequently ChIP-seq, for CTCF on pro-B cells and pre-B cells and indeed we found that there were many sites bound in the Igh and Ig kappa light chain loci (35, 36). However, it appeared from the ChIP-chip and from ChIP/qPCR that the CTCF binding at the Igh locus, although lymphoid specific, showed limited lineage- and stage-specificity (i.e., similar numbers in pro-B cells, pre-B cells, and thymocytes) (35). By contrast, we showed that CTCF binding demonstrated more stage-specificity at the Igκ locus. Thus, CTCF binding, by itself, cannot explain locus contraction, even though it contributes to the 3D conformation of the contracted Igh locus as determined by knockdown of CTCF in pro-B cells (36). However, we also performed ChIP-chip and ChIP/qPCR for Rad21, a component of the cohesin complex, and the pattern of Rad21 binding showed more developmental stage-specificity in both Ig loci (35).

In the current study, we greatly extend this analysis by presenting a detailed analysis of our ChIP-seq data of the pattern of CTCF and Rad21 binding in all of the Ig and TCR loci at the two stages of B cell development and two stages of T cell development when the various AgR loci undergo rearrangement. In addition, we also analzyed pre-pro-B cells, mature B and T cells, ES cells, and murine embryonic fibroblasts (MEF), in order to assess the extent of lineage- and stage-specificity of CTCF and cohesin binding at AgR loci. Although much of the CTCF binding in the large V region part of the AgR loci is lymphoid specific, our data presented here show that the extent of lineage (T vs. B lineage precursors) and developmental stage-specificity (pro-B vs. pre-B, DN vs. DP) of CTCF and cohesin binding varies significantly from locus to locus, with the Igκ locus being the only one of the 6 AgR loci with clear lineage- and stage-specificity of the onset of most CTCF and cohesin binding. Also, we show that the pattern of CTCF and cohesin binding observed in the most mature B or T cell precursor population is maintained in mature B or T cells, respectively. MEFs show many fewer sites of binding while ES cells are intermediate. In contrast to the large V gene portion of each locus, the CTCF sites between the V and D/J genes and near enhancers tend to display conserved CTCF and cohesin binding among all of the cell types examined including non-lymphocytes. We hypothesize that the acquisition of binding of many CTCF/cohesin complexes within the V gene portion of each of the four large AgR loci establish many long-range loops, and provide a semi-contracted locus upon which transcription factors can act to direct full locus contraction so that V genes throughout each locus will have approximately equal opportunity to rearrange to create a diverse set of V(D)J rearrangements.

## Materials and Methods

### Cell Types Being Analyzed

For all ChIP-seq analyses of AgR loci in lymphocyte progenitors, we use RAG1-deficient cell lines so that the AgR locus remains intact, unperturbed by V(D)J rearrangements that will result in unique deletions in each individual B or T cell precursor. RAG deficiency also provides a precise genetic block in differentiation, so the cell populations are free of contamination from later stages of differentiation. All CD19^+^ cells from BM of RAG1^−/−^ mice are pro-B cells. All CD19^+^ cells from BM of RAG^−/−^ mice bearing a rearranged IgM transgene are pre-B cells (37). Likewise, thymocytes from RAG^−/−^ mice are all CD4^−^CD8^−^ “double-negative” (DN) cells, and thymocytes from RAG^−/−^ mice bearing a Vβ transgene are all at the subsequent stage of “double-positive” (DP) cells. IL7-cultured pro-B cells were obtained from day 7 cultures of RAG1^−/−^ pro-B cells cultured in media containing supernatant from J558 cells stably expressing IL7 as previously described (38). All mice were on the C57BL/6 background. All animal work is approved by The Scripps Research Institute’s IACUC and follows their guidelines.

### ChIP/qPCR

ChIP was done with antibodies against H3K4me3 (Active Motif, Carlsbad, CA, USA), H3ac (EMD Millipore, Billerica, MA, USA), and H3K4me1 (Abcam) as previously described (39). Primers are listed in Table S1 in Supplementary Material.

### Gene Expression Analysis

RNA was extracted from cells using TRIzol (Life Technologies, Carlsbad, CA, USA), and cDNA was made with QuantiTect Reverse Transcription kit, and genomic DNA was elminated using genomic DNA wipeout buffer (Qiagen, Valencia, CA, USA).

### ChIP-seq

ChIP-seq for CTCF and Rad21 were performed as previously described (36). CTCF and Rad21 ChIP-seq on pro-B cells were previously published (36), as were CTCF ChIP-seq on DN and DP (40) and CTCF and Rad21 on mature B and T cells (41, 42). Pre-pro-B cells were obtained from long-term culture of E2A^−/−^ progenitors and the ChIP-seqs were from GEO GSM987801 and GSM987802 (43). The CTCF ChIP-seq for ES and MEF were GEO (GSM723004, GSM723008) (44). Rad21 ChIP-seq was only available for ES cells (GSM824847, GSM824848) (45), but not for MEF, so we also have included ChIP-seqs for another cohesin component, Smc1, for ES cells (GSM560341, GSM560342) and MEF (GSM560355, GSM560356) (46). All ChIP-seq were mapped to the reference genome of C57BL/6, mm9. All GEO accession numbers, including the new entries, are listed in Table S2 in Supplementary Material.

We utilized ChIP-seq performed in our lab over a 4-year period for pro-B cells, pre-B cells, DN, and DP thymocytes for CTCF and Rad21. The ChIP-seq for pre-pro-B cells, mature T and B cells, MEF and ES cells were obtained from GEO. In order to make the Genome Browser files from different labs and over different times and platforms comparable, we zoomed out to view 90 Mb regions of the genome on a few chromosomes, and adjusted the scale to make them equivalent in height (Figures S1A,B in Supplementary Material). This was done since we know that over half of CTCF sites are constant among different cell types, and that TADs, which are bounded by CTCF sites, are also relatively conserved across cell types (8, 15).

### Analysis of ChIP-seq Data

Preliminary quality control over raw sequence data was performed with FastQC 0.11 (47). Duplicate reads were removed before mapping, and TruSeq adapter sequences were removed with the HOMER trim tool (48). Experimental and input control fastq tags were aligned to the mouse reference genome (mm9) using Bowtie 1.1.2 (alignment parameters: -a -v 2 -m 3 —best –strata) (49).

Signal tracks normalized to 1M reads were generated from alignment (bam) files using MACS2 (50) according to the procedure described in Ref. (50) (parameters: macs2 callpeak -B —nomodel —extsize 147 —SPMR -g mm).

Confident peaks were called using MACS (v1.4.2) (50), with a false-discovery rate (FDR) ≤1% and the default *p*-value (1E-5) as specified in Ref. (51). Downstream analysis and manipulation of the data, including annotation of peaks, motif finding, quantification of data at peaks/genomic regions, and overlap analysis, were performed with HOMER 4.8 (48) and R/Bioconductor (52). Custom code is available upon request.

In particular, peaks were annotated with genomic features obtained from RefSeq release 66 through homer.ucsd.edu/homer/data/genomes/mm9.v5.7.zip. Peak overlaps were computed using the HOMER mergePeaks tool (parameter used: -d for literal overlaps).

The above pipeline was used to uniformly process all of our ChIP-seq data and raw sequencing data obtained from GEO. For the Rad21 and Smc1 (another cohesin subunit) ChIP-seq on ES and MEF, we used the called peaks provided by the authors.

### Analysis of Orientation for CTCF Sites

In order to assess directionality of CTCF-binding sites, we devised the following procedure:

(1) Assemble a library of CTCF motifs. A total of nine CTCF motifs were considered—five motifs retrieved from InsulatorDB, and four motifs detected in pre-B and pro-B ChIP-seq with HOMER findMotifsGenome.pl. (three known motifs and one *de novo*) (Table S3 in Supplementary Material). InsulatorDB motifs were converted to motif matrices (input for subsequent HOMER processing steps) using HOMER seq2profile tool with 0 mismatches. Motif matrices for HOMER-detected motifs were downloaded directly from the motif finding report. Finally, a custom motif file was created following HOMER guidelines.
(2) Run annotatePeaks.pl (HOMER) on confident ChIP-seq peaks in a specific region (bed file) against the above library of CTCF motifs (e.g., for Igκ it was CTCF pre-B ChIP-seq, limited to the Igκ locus). This will generate a peak annotation file with instances of CTCF motifs found within the confident peaks in the region.
(3) For each peak (i.e., a row in the above annotation file) annotated to motif(s), assign a (+) (forward) or (–) (reverse) orientation to each motif, by checking the strand on which the motif is detected. If multiple motifs are detected, take the consensus sign. Ambiguous cases are discarded.
(4) Using the data in the annotation file, assemble a bed file with strand (orientation) information.

## Results

### Optimization of Mapping Parameters for AgR Loci

We have found that the common method of analyzing ChIP-seq data by mapping only unique reads is not appropriate for AgR loci which have arisen by extensive gene duplication of individual V genes. It is especially important for the TCRα/δ locus, which has a relatively recent triplication of a large portion of the locus in C57BL/6 mice, the genome on which all of these data are mapped (53). This became obvious when we mapped CTCF sites, and observed that the two more distal duplications (the “d” and “n” repeats) appeared not to have CTCF bound to them despite the fact that all three copies are extremely similar. We varied the parameters of the mapping, and found that the parameter v2m3, which allows retention and mapping of reads which map up to three different locations within the AgR locus, allows for accurate mapping (Figure S2A in Supplementary Material). This was verified by ChIP/qPCR using PCR primers that can distinguish the 3′ proximal repeat from the d/n repeats, thus assaying the three triplicated regions. As can be seen in Figure S2B in Supplementary Material, both the 3′ and d/n repeats bind CTCF. It can be seen that even in loci such as the Igh locus where the mapping difference between parameters are not as dramatic as at the TCRα/δ locus, the number of reads decreased somewhat at v2m2 and more at v2m1. In particular, the distal half of the Igh locus, which harbors the large VhJ558 family, displays fewer reads at v2m1 (Figure S2C in Supplementary Material). Although other AgR loci tend to display greater V segment sequence divergence than in the TCRα/δ locus, the v2m3 parameter is beneficial for all ChIP-seq analyses within multi-V gene AgR loci, and it was used for all of the analyses presented here.

### D-J-C and Enhancer-Proximal CTCF and Cohesin Binding Is Generally Not Lineage-Specific

To determine the lineage and stage-specificity of CTCF and cohesin binding, we performed ChIP-seq for CTCF and Rad21 (a subunit of cohesin) for two sequential stages in B lymphocyte differentiation (pro-B and pre-B) and two stages in thymocyte differentiation (DN and DP). We compared these data to published data for binding in ES cells and MEFs. The four largest AgR loci (Igh, Igκ, TCRβ, and TCRα/δ) have a similar organization with a large number of V genes spread over a major percentage of the loci, followed by the (D)J-C genes tightly clustered at one end of the locus. The much smaller Igλ and TCRγ loci have a very different organization and many fewer V genes, and will be discussed later. All four large AgR loci have 1–4 CTCF bound sites between the V genes and the D and/or J genes, and the majority of these sites in the Igh, Igκ, and TCRα/δ loci are bound by CTCF and cohesin not only in all four lymphocyte subsets but also in MEF and ES cells. Nevertheless, in some cases, such as Cer and Sis in the Vκ-Jκ intervening region (54, 55), only one of the two sites may be bound in all cell types (Table 1). By contrast, in the TCRβ locus, occupancy of CTCF and cohesin at the CTCF site called “5′PC” located 27.6 kb upstream of the Dβ1.1 gene (56), the weak CTCF site near the Dβ1.1 gene, and the CTCF site between Eβ and Vβ31 is predominantly restricted to DP and DN thymocytes.

**Table 1:** CTCF and cohesin binding in D-J-C-enhancer regions: lineage- and developmental stage-specificity.

CTCF locus	Between V and D/J	Enhancer	# CTCF sites	Conserved in all lymphocyte subsets?	MEF	ES
Igh	IGCR1		2	Yes	Low (site #2 only)	Yes
		3′RR	9	Yes	Yes	Yes

Igκ	Cer		2	Yes but only site #1 in DN, DP	Yes	Yes
	Sis		2	Yes	Site #2 only	Site #2 only
		none

TCRα/δ	TEA		1	Yes	Yes	Yes
		Eα	2	Yes	Yes	Yes

TCRβ	TCRβ 5′ PC (between V and D) TCRβ 3 kb up from Dβ1.1		1
			1	DP, DN (tiny peak)	No	No
		Eβ/Vβ31	1	DN, DP, pro-B only	No	Low

**Cohesin locus**	**Between V and D/J**	**Enhancer**	**# CTCF sites**	**Conserved in all lymphocyte subsets?**	**MEF**	**ES**

Igh	IGCR1		2	Only #2 in DN, pre-pro-B	Low (site #2 only)	Yes
		3′RR	9	Fewer in DP, DN	No	Yes

Igκ	Cer		2	Yes, but only site #1 in DN, DP; none pre-proB	No	Yes
	Sis		2	pro-B, pre-B, DP only	Very low	Yes
		none				

TCRα/δ	TEA		1	Low in proB, preB, not in pre-pro-B	Yes	Yes
		Eα	2	Yes	Yes	Yes

TCRβ	TCRβ 5′ PC (between V and D)		1	DP, DN only	No	No
	TCRβ 3 kb up from Dβ1.1		1	DP, DN (tiny peak)	No	No
		Eβ/Vβ31	1	DN, DP only	Yes	Yes

*All peaks are ones identified by the peak-calling program MACS*.

### Variable Lineage-Specificity of CTCF and Cohesin Binding in the V Gene Portions of AgR Loci

**Figure 1:**
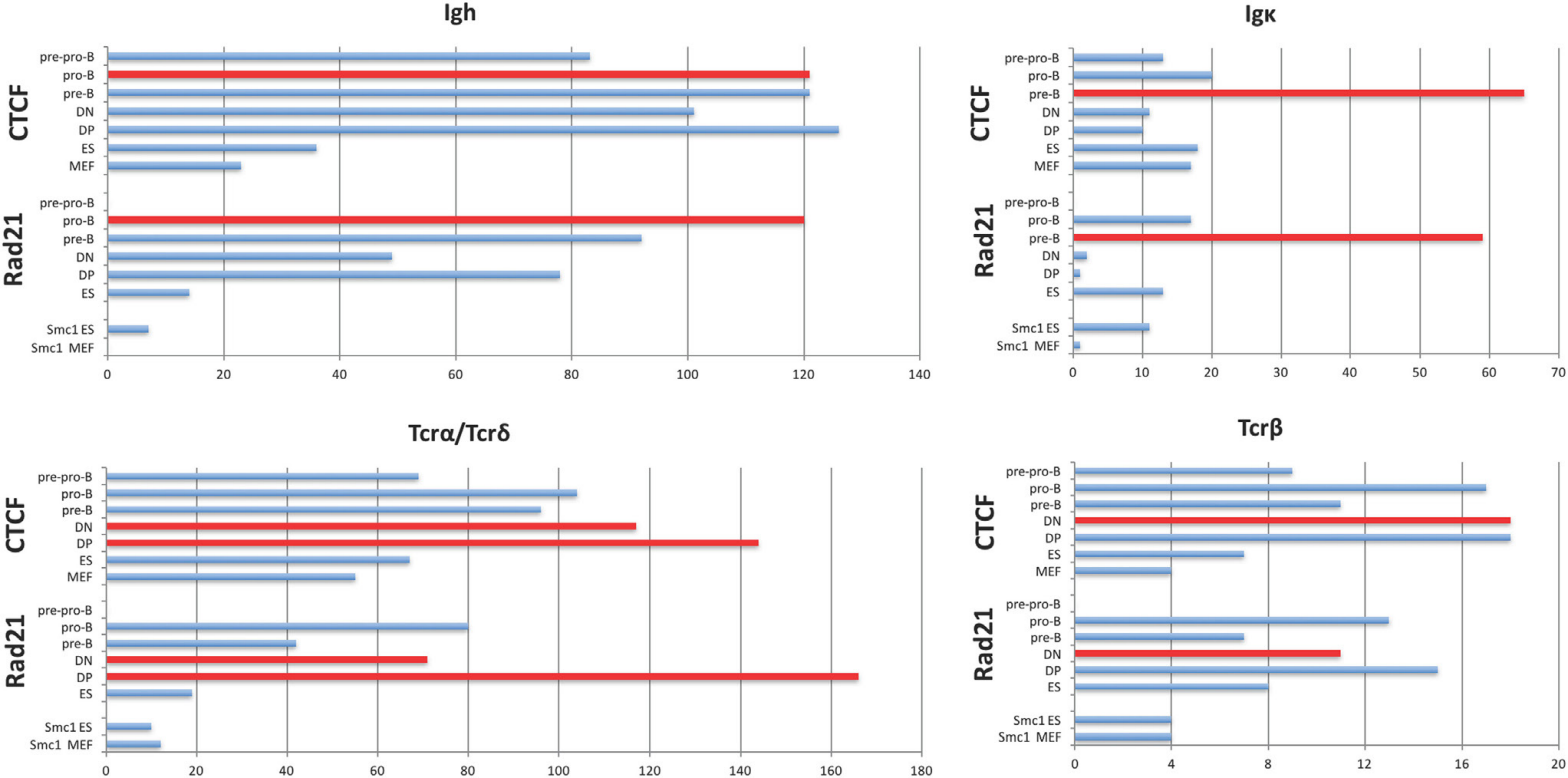
Plots of the number of significant peaks within the V gene-containing portion of each AgR locus called by MACS from ChIP-seq of pre-pro-B cells, pro-B cells, pre-B cells, double-negative (DN) and double-positive (DP) thymocytes, and murine embryonic fibroblast (MEF) and ES cells. The Rad21 and Smc1 peaks for ES and MEF were obtained from GEO, and the number of called peaks were obtained from GEO files. The cell type which is rearranging the locus being analyzed is plotted in red. The TCRα/δ locus rearranges at two stages since the TCRδ genes rearrange in DN thymocytes, and the TCRα genes rearrange in DP thymocytes. Statistics are shown in Tables S4 and S5 in Supplementary Material.

In contrast to the fairly uniform binding of CTCF/Rad21 in the D-J-C regions among diverse cell types, CTCF and Rad21 binding within the large V portion of these loci shows greater lymphocyte specificity (Figure 1). The Igh, TCRβ, and TCRα/δ loci show remarkably similar numbers of sites of CTCF binding throughout all stages of B and T progenitor populations, but many fewer sites in MEF and ES cells (Figure 1; Table S4 in Supplementary Material). Thus, they show lymphocyte-specificity, but among lymphocytes, lack lineage- and developmental stage-specificity. Pre-pro-B cells (E2A^−/−^ early progenitors) show many fewer bound CTCF peaks, suggesting that entry into the B progenitor stage is important for the initiation of CTCF binding in Ig loci. Only the Igκ locus shows marked lineage- and stage-specificity for CTCF binding.

The number of V genes in each locus, the number of CTCF sites that are bound in the V gene portion of each locus at the stage of its rearrangement, and the location of those bound CTCF sites relative to the V genes coding regions are shown in Table 2. The number of bound sites does not directly correlate with the size of the locus. The number of CTCF or Rad21 bound peaks within the V region portion of the 2.8 Mb Igh and the 1.8 Mb TCRα/δ loci are well over 100 (Figure 1; Table 2). The 3.2 Mb Igκ locus has only 65 bound CTCF sites, and the much smaller 666 kb TCRβ locus has 18 bound CTCF sites. A hypergeometric test shows significant enrichment of the number of bound sites in the AgR loci in the various cell types studied compared to genome-wide binding (Table S5 in Supplementary Material). 76–95% of peaks contain consensus motifs within each locus (Table 3).

**Table 2:** Bound CCCTC-binding factor (CTCF) in V gene portions of all AgR loci.

Locus	Total number of V genes	# Functional V genes	Size of entire locus	# Bound CTCF within V gene locus	Location of CTCF sites relative to V genes
I_g_H	195	113	2.8 Mb	121	Within 100 bp of the RSS for most genes in the Vh families: 7183, Q52, S107, X24, Vh11, Vh12, Vh10, J606; intergenic for all the other Vh families
I_g_κ	140	125	3.2 Mb	65	Most intergenic, only a few within 4–8 kb upstream of promoter
I_g_λ	3	3	233 kb	3	Upstream of each V gene
TCRβ	33	22	666 kb	18	Several at/near promoters; 2 near RSS, several have none
TCRα/δ	131	109	1.8 Mb	144	Many upstream of V gene promoter, some intergenic
TCRγ	7	7	174 kb	2	Flanking 2 of 3 V clusters

*The number of CTCF sites bound is for the cell type in which that locus rearranges*.

**Table 3:** CTCF peaks with consensus motifs within V gene portion of each locus.

Region analyzed	ChIP-seq analyzed	Total # peaks (MACS)	# Peaks with one or more of the CTCF motifs	% of MACS peaks that matched at least one of nine CTCF PWM
genome wide	pre-B	42,250	34,560	81.8
Vh in Igh	pro-B	121	115	95.0
Vκ in Igκ	pre-B	65	50	76.9
Vβ in TCRβ	DN	18	14	77.8
Vα/δ in TCRαδ	DP	144	114	79.2

With the exception of the TCRβ locus, the large loci show more lineage- and stage-specificity in binding of the cohesin subunit Rad21 (Figure 1). As with CTCF binding, the Igκ locus shows the greatest lineage- and stage-specificity, and again, MEF and ES cells show the least amount of Rad21 bound. However, it should be noted that our lymphocyte and non-lymphocyte data for Rad21 and Smc1 (another cohesin subunit) may not be directly comparable because we used MACS to calculate Rad21 peaks from all lymphocyte subsets whereas we plotted peak calls obtained by the authors for Rad21 and Smc1 in ES cells and MEFs. Nonetheless, cohesin binding appears to contribute greater lineage-specificity than CTCF binding at AgR loci.

### Lineage- and Stage-Specificity of CTCF and Cohesin Binding Intensity in AgR Loci

Although the number of MACS called CTCF peaks in a locus is often similar among the different stages of lymphocyte differentiation, we observed substantial developmental stage-specificity in the abundance of reads (heights of peaks) of CTCF and cohesin binding at these sites. Often binding was highest in the developmental stage during which rearrangement normally occurs. The predominant reason for a higher peak at any specific location in one cell type vs. another is that a larger proportion of the cells used for the ChIP-seq lysate had CTCF (or Rad21) bound at that site. Within a locus, one reason for different heights of the various CTCF peaks could also be that a different proportion of cells had CTCF bound at the different sites, or it could reflect differences in the binding strength of CTCF to the individual sites, since the motif for CTCF is quite variable (57).

We examine each of the four large AgR loci separately in the sections below. The analysis of the two smaller AgR loci that do not have the same V, D, and J gene organization pattern will be presented later.

#### Igh Locus

**Figure 2:**
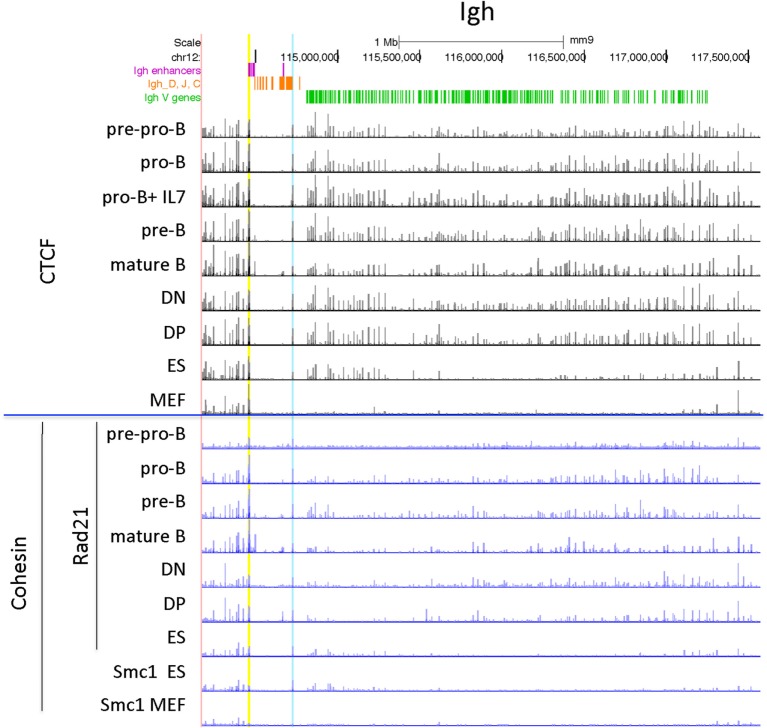
Genome Browser views of CCCTC-binding factor (CTCF) and cohesin subunit (Rad21 or Smc1) ChIP-seqs for the Igh locus. IGCR1 (the set of two CTCF sites upstream of DFL16.1) is highlighted in blue and the set of nine CTCF sites in the 3′ regulatory region are highlighted in yellow.

The Igh locus rearranges in the pro-B cell stage. T cells display some Dh-Jh rearrangements which may have occurred before the B and T cell lineages have bifurcated from their common progenitor (59). The 121 CTCF sites within the 2.5 Mb of the V gene part of the Igh locus show a similar pattern of peak heights of CTCF binding throughout the locus for the lymphocyte subsets examined, although the peak heights are lower overall in the pre-pro-B cells (Figure 2). By contrast, the MEFs display very little binding. The ES cells show a pattern of CTCF and cohesin binding similar to the lymphocytes at the Jh-proximal ~800 kb whereas the remaining Jh-distal ~1.7 Mb show few if any occupied CTCF sites in ES cells. The Rad21 binding pattern is similar for the pro-B, pre-B cells and mature B cells, but shows low binding in only in the most Jh-proximal part of the locus in MEF and ES cells. Pre-pro-B cells show little Rad21 binding anywhere within the locus.

#### Igκ Locus

**Figure 3:**
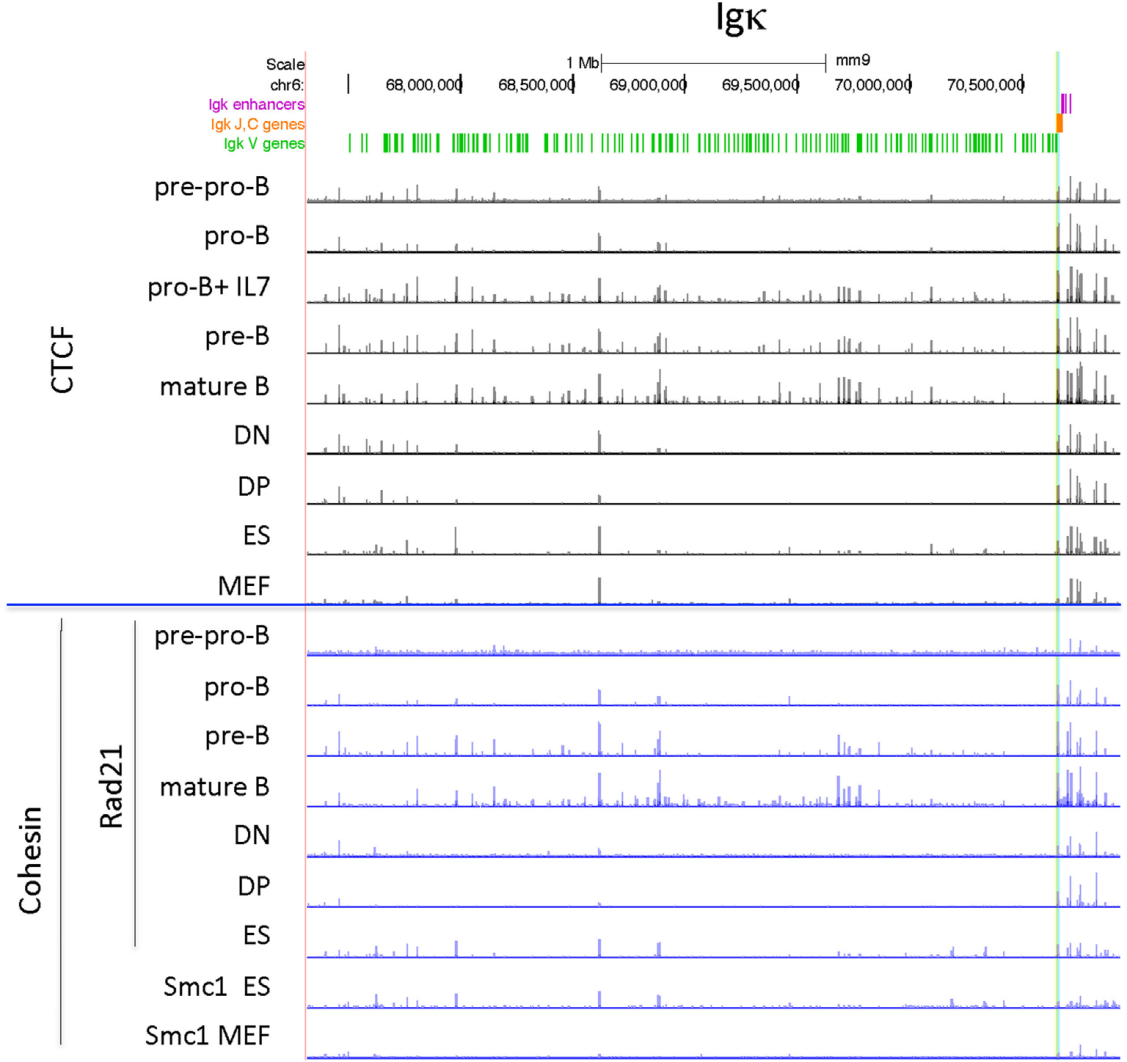
Genome Browser views of CCCTC-binding factor (CTCF) and cohesin subunit (Rad21 or Smc1) ChIP-seqs are plotted for the Igκ locus. The two CTCF sites in Cer are highlighted in yellow and the two CTCF sites in the adjacent Ser region are highlighted in blue.

The Igκ locus rearranges in pre-B cells and displays maximal CTCF and cohesin binding at that stage (Figure 3). Only one strong binding site 2 Mb away from the Jκ regions is present in all cell types examined, but the majority of the peaks show extensive specificity. Pro-B cells display lower level CTCF binding to several sites in the middle and Jκ-distal end of the locus, but very little binding at all to the entire proximal half of the V portion of the locus. Other lymphocyte subsets and ES cells display only a few Jκ-distal CTCF-binding peaks. There is little to no Rad21 binding in MEFs and pre-pro-B cells, and very few sites bound in DN and DP thymocytes. ES cells have some peaks for both Rad21 and Smc1 cohesin subunits, and pro-B cells have a few small peaks. Thus, the Igκ locus shows greater lineage- and stage-specificity of CTCF and cohesin binding than any of the other AgR loci.

**Figure 4:**
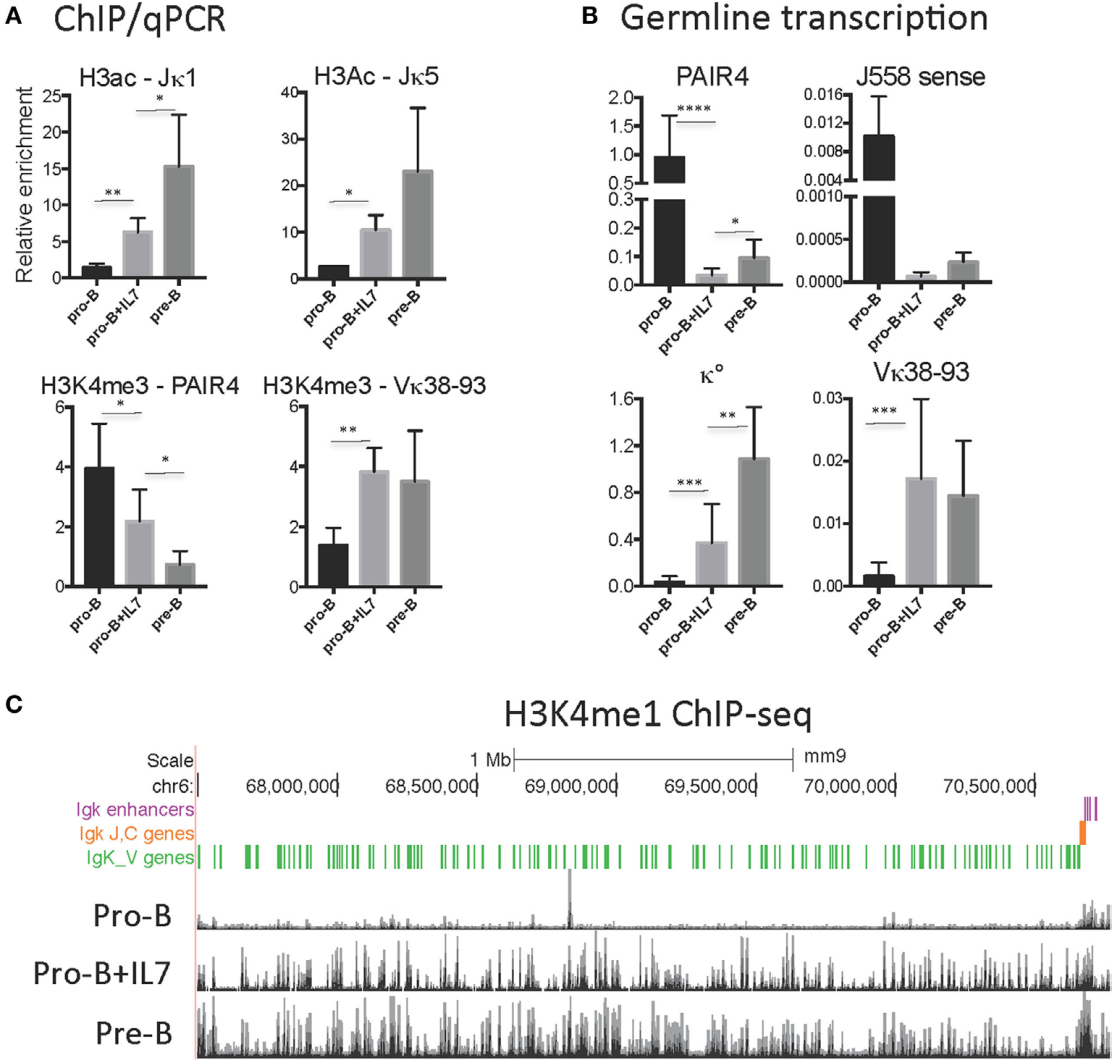
**(A)** H3K4me3 and H3ac ChIP/qPCR on RAG1^−/−^CD19^+^ pro-B cells, RAG1^−/−^IgH Tg^+^ CD19^+^ pro-B cells, and RAG1^−/−^ pro-B cells cultured with IL7. **(B)** Relative levels of transcription of ncRNA (germline transcription) in the Igh (PAIR and J558 sense) and Igκ (κ° and Vκ38-93) loci in RAG1^−/−^ CD19^+^ pro-B cells, RAG1^−/−^IgH Tg^+^ CD19^+^ pre-B cells, and RAG1^−/−^ pro-B cells cultured with IL7. **(C)** H3K4me1 ChIP-seq on RAG1^−/−^ CD19^+^ pro-B cells (38), RAG1^−/−^ pro-B cells cultured with IL7 (58), and RAG1^−/−^IgHTg^+^ CD19^+^ pre-B cells throughout the Igκ locus. Significance determined with Mann–Whitney test. * is <0.05, ** is <0.01, *** is <0.001, and **** is <0.0001.

Culture of pro-B cells in IL7 for several days is often done to obtain large numbers of pro-B cells for ChIP or ChIP-seq (58, 60). However, we found that the CTCF-binding pattern of IL7-cultured RAG^−/−^ pro-B cells resembles that of pre-B cells, not pro-B cells, in that all of the binding sites in the proximal half of the locus are now strongly binding CTCF (Figure 3). To further address the nature of these cells, we analyzed the epigenetic markers H3ac and H3K4me3 at select sites within the Igh and Igκ loci by ChIP/qPCR, and also analyzed the production of ncRNA (also known as “germline transcription”) (Figure 4). Within the Igh locus, pro-B cells display extensive ncRNA at three PAIR elements in the distal part of the locus (61), and ChIP/qPCR shows a 10-fold reduction of this transcription in pre-B cells, and even greater reduction in IL7-cultured pro-B cells (Figure 4B). Likewise the level of ncRNA over the VhJ558 genes (62) is substantially lower in both pre-B and IL7-cultured pro-B cells. The extent of H3K4me3 at PAIR4 in IL7-cultured pro-B cells is intermediate between that in pro-B and pre-B cells. ChIP-seq for H3K4me1, the epigenetic mark of enhancers, shows that although pro-B cells have very few sites of H3K4me1 in the Vκ locus in pro-B cells, IL7-cultured pro-B cells resemble pre-B cells in that they have many regions of H3K4me1 bound throughout the Vκ locus (Figure 4C). Transcription of the κ° ncRNA (63), which runs through the Jκ and Cκ genes is greatly increased in pre-B cells, and is intermediate in the IL7-cultured pro-B cells compared to pro-B cells (Figure 4B). Likewise, the extent of H3ac at the Jκ genes is greatly increased in pre-B cells compared to pro-B cells and is intermediate in IL7-cultured pro-B cells (Figure 4A). There is a relatively high level of ncRNA through the Vκ38-93 gene in pre-B cells but not pro-B cells, and the IL7-cultured pro-B cells resemble pre-B cells in both ncRNA transcription as well as in the H3K4me3 profile. Thus, for epigenetic marks, germline transcription, and CTCF binding, IL7-cultured pro-B cells resemble pre-B cells more than pro-B cells for many characteristics. Thus, previous published data on IL7-cultured pro-B cells should be interpreted with caution.

#### TCRα/δ Locus

**Figure 5:**
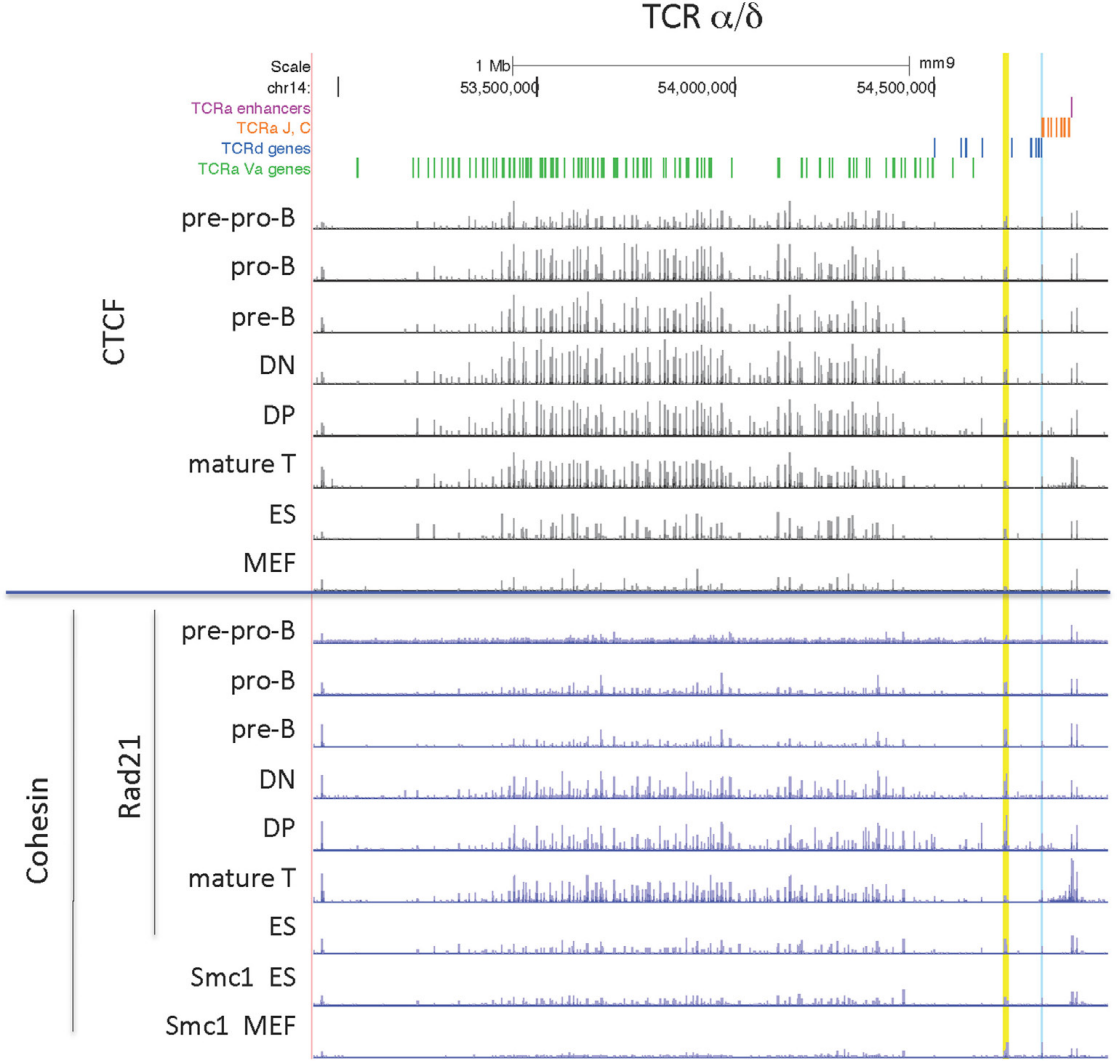
Genome Browser views of CCCTC-binding factor (CTCF) and cohesin subunit (Rad21 or Smc1) ChIP-seqs are plotted for the TCRα/δ locus. The TEA element is highlighted in blue and INT1/INT2 is highlighted in yellow.

The organization of this locus is by far the most complex since it contains all the genes for both the alpha and delta chains of the TCR with TCRδ rearrangement occurring at the DN stage of thymocyte development, and TCRα rearrangement in the subsequent DP stage of development. However the pattern of CTCF binding at the TCRα/δ locus is very similar in all lymphocyte subsets, with the exception of particularly low level binding in pre-pro-B cells (Figure 5). ES cells also have many sites bound by CTCF, and only MEFs have noticeably smaller CTCF peaks. By contrast, high level Rad21 binding is restricted to the two thymocyte subsets and mature T cells, and there is almost no cohesin bound in MEF or pre-pro-B cells.

#### TCRβ Locus

**Figure 6:**
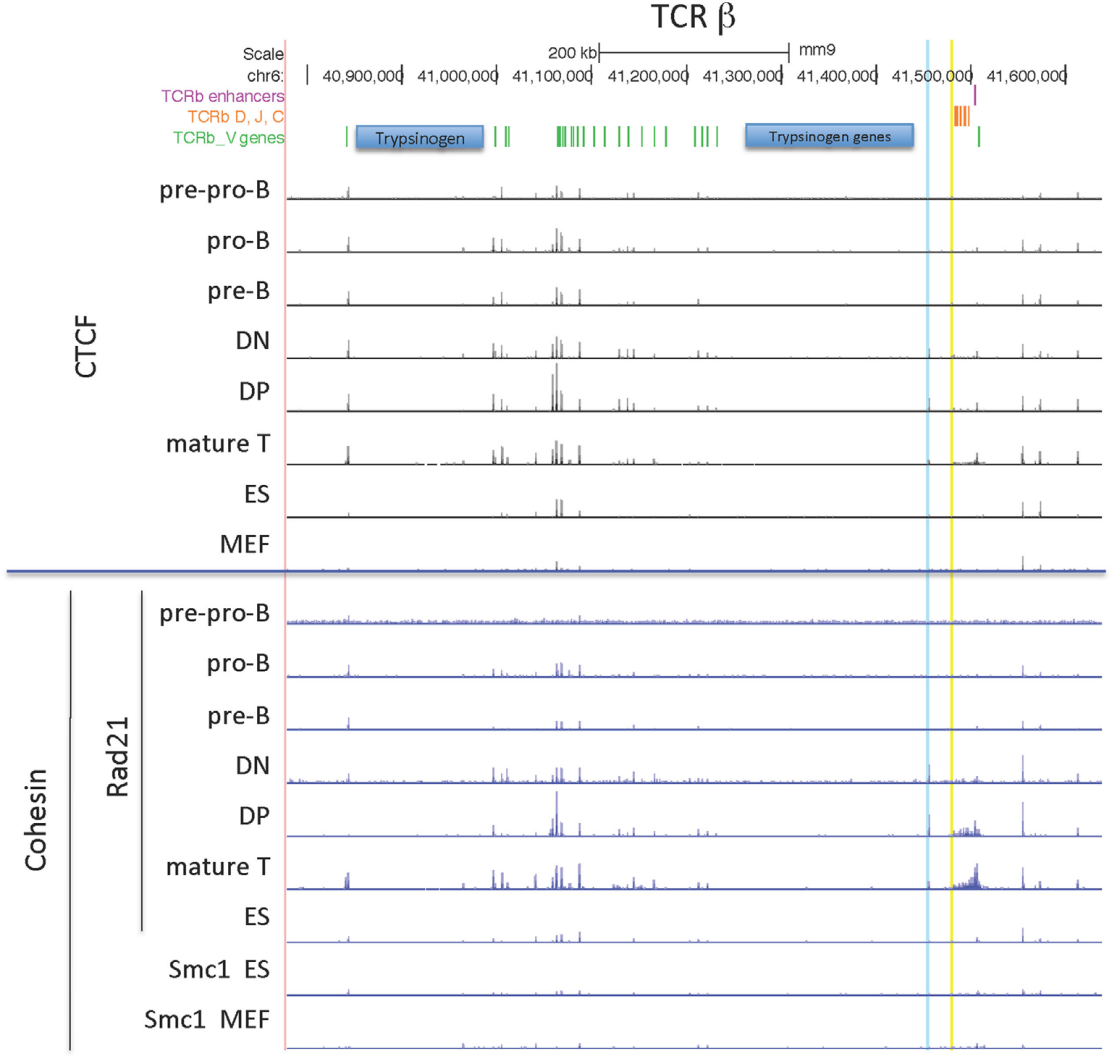
Genome Browser views of CCCTC-binding factor (CTCF) and cohesin subunit (Rad21 or Smc1) ChIP-seqs are plotted for the TCRβ locus. 5′PC is highlighted in blue and the low CTCF site near Dβ1 is highlighted in yellow.

The TCRβ locus is the smallest of the 4 large AgR loci, with all but 2 of its 22 functional V genes located in a compact 235 kb region. CTCF binding throughout the locus is similar in all lymphocyte subsets, but much less binding is found in ES cells and MEFs (Figure 6). Rad21 binding is highest in T cell subsets, but there is low level binding in B cell progenitors as ES cells.

### CTCF Orientation within the AgR Loci

It has been documented that 65–90% of long-range CTCF–CTCF interactions take place between CTCF sites present in convergent orientation (facing each other) (10, 64). The boundaries of TADs have a very high percentage of convergently oriented CTCF loops, probably due to the process of loop extrusion (20). However, there is more variable orientation of pairs of CTCF sites within TADs (13, 65). Since the many CTCF sites within the AgR loci could serve to create the rosette structure that the AgR loci are thought to adopt, and since they could also aid in bringing the many V genes, spread out over Mb in some cases, into close proximity to D-J genes, the orientation of CTCF sites within the AgR loci is of critical importance for predicting the role and location of CTCF–CTCF loops (28). We, therefore, compiled 9 CTCF motifs as described in Section “Materials And Methods,” and determined the orientation of all CTCF sites identified by any of those motifs.

**Figure 7:**
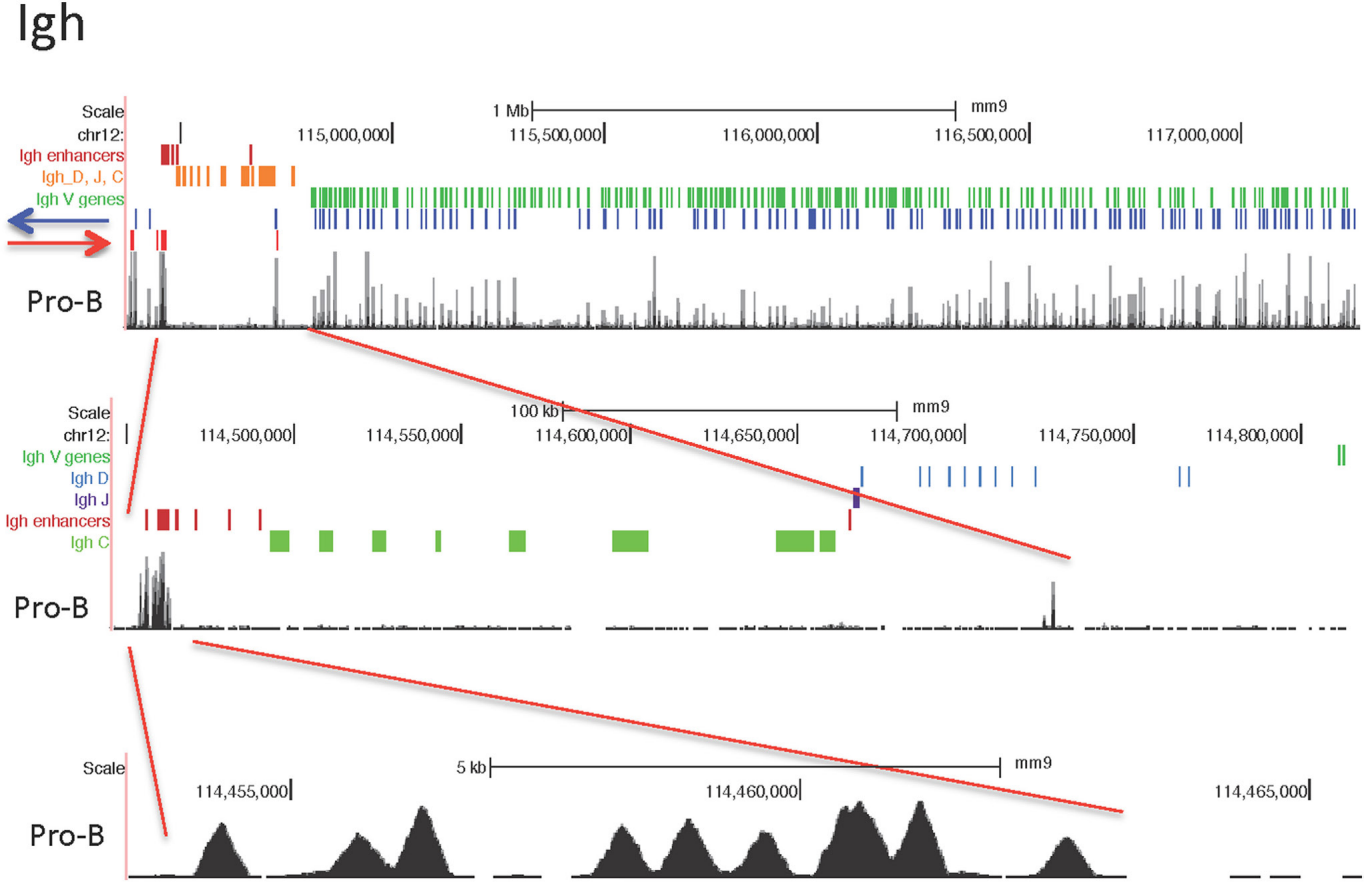
Orientation of CCCTC-binding factor (CTCF) sites in the immunoglobulin heavy chain (Igh) locus, with close-up of the D-J-C-enhancer region in the middle panel, and close-up of the 3′ regulatory region in the bottom panel.

**Figure 8:**
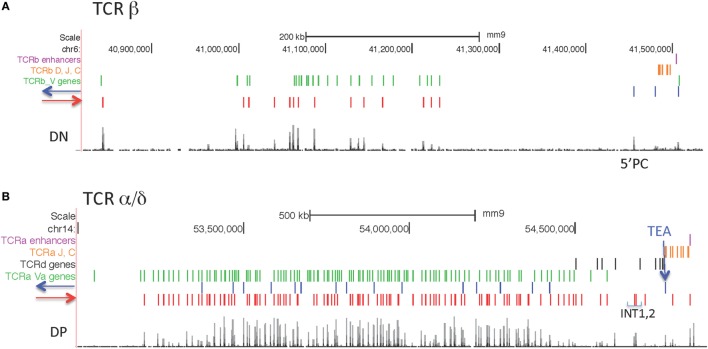
**(A)** Orientation of CCCTC-binding factor (CTCF) sites in the TCRβ locus. **(B)** Orientation of CTCF sites in the Tcrα/δ locus.

Two of the large AgR loci, Igh and TCRβ, have rather simple CTCF orientation patterns, with all bound CTCF sites in the large V gene portion of the loci pointing toward the D-J-C regions, and most of the CTCF sites in the D-J-C-enhancer regions oriented toward the V region CTCF sites (66) (Figures 7 and 8A). By contrast, the two other large AgR loci, Igκ and TCRα/δ, have complex patterns of CTCF orientation, with many of the sites within the V portions of the loci pointing away from the D-J-C regions as well as many oriented toward the D-J-C regions, as described below.

#### TCRα/δ Locus

CTCF sites are located in both orientations in the V part of the locus, although the majority of sites (129/144) are pointing toward the J-C regions (Figure 8B) as previously observed (67). The CTCF site at the TEA promoter is an important regulatory element located upstream of the 61 Jα gene segments (68, 69), and it is oriented toward the V genes. The next CTCF site with the same orientation is just upstream of the V gene Trav17. This CTCF near Trav17 is oriented to make long-range loops with any of the many upstream CTCF sites throughout the V region part of the locus. Two prominent CTCF sites upstream of Trdv2-2 are oriented toward TEA, and have been demonstrated interact with TEA (67). Deletion of those two CTCF sites, called INT1 and INT2, results in altered TCRδ and TCRα V gene usage (67).

#### Igκ Locus

**Figure 9:**
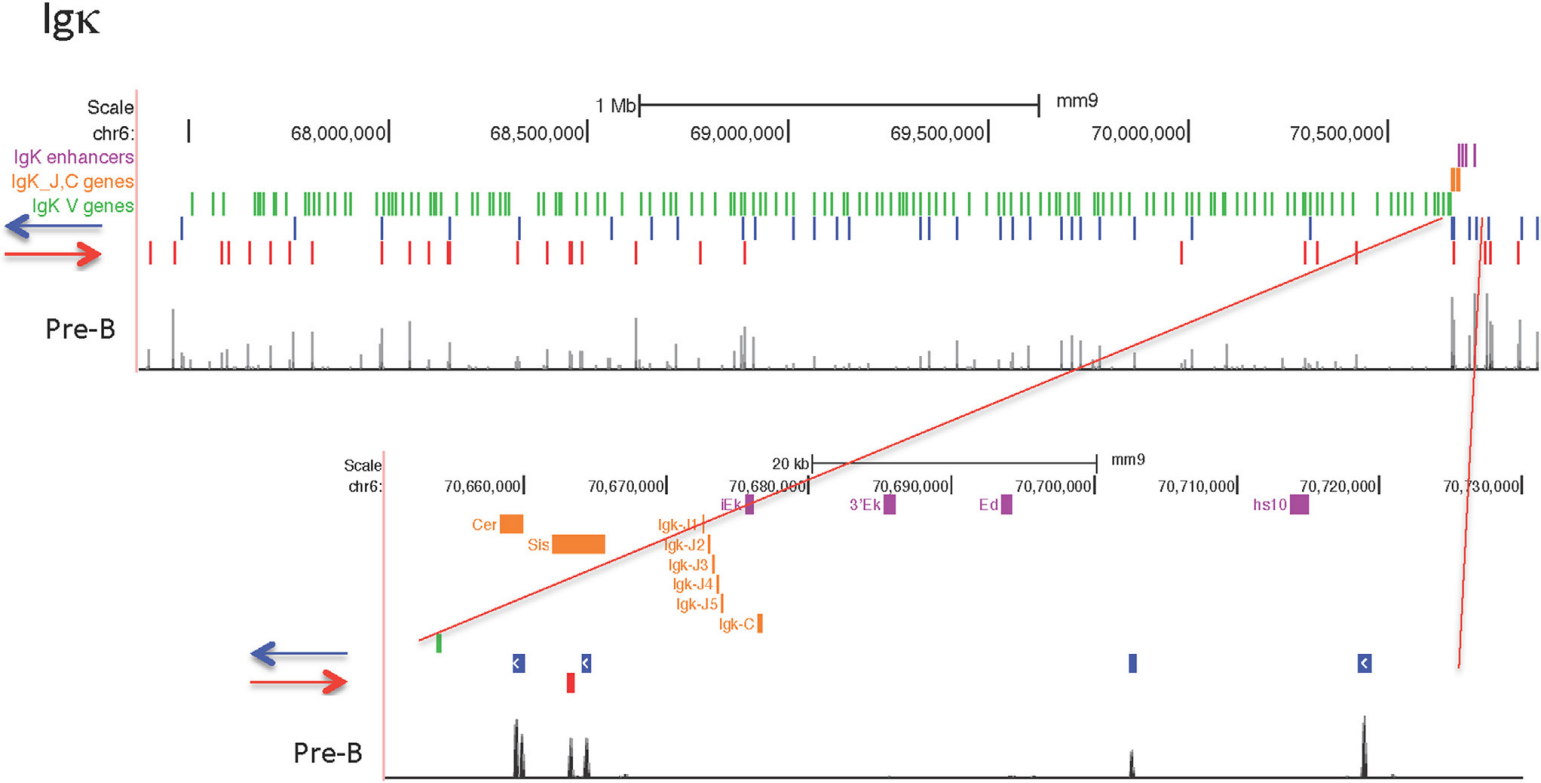
Orientation of CCCTC-binding factor (CTCF) sites in the Igκ locus, with close-up of the Cer, Sis, Jκ-Cκ-enhancer region.

The Igκ locus has four CTCF sites in between the V genes and the J genes. The set of two sites closest to the V genes, called Cer, plays an important role in locus conformation and regulation, and both CTCF sites are oriented toward the V genes (55). The set of two CTCF sites closest to the Jκ genes, called Sis, has a less prominent role in locus regulation (54). One of those CTCF sites in Sis is orientated toward the V genes and the other is in the opposite orientation. The CTCF sites in the 3.1 Mb V region portion of the locus are present in both orientations (Figure 9). The majority of sites in the J-proximal half of the locus are pointed away from the CTCF sites in the V-J intervening region. Thus, these CTCF sites are not oriented to participate in looping to convergently oriented CTCF sites that would bring proximal V genes closer to the J genes. By contrast, the majority of CTCF sites in the distal half of the locus are oriented toward the J genes, and thus could potentially interact in convergent orientation with the CTCF sites in Cer and Sis in the V-J intervening region. Unlike all other AgR loci, approximately half of the Vκ genes are in the opposite transcriptional orientation to the J gene segments and so rearrange by inversion. However, there is no correlation between the orientation of CTCF sites and the transcriptional orientation of the Vκ genes.

#### TCRγ Locus

**Figure 10:**
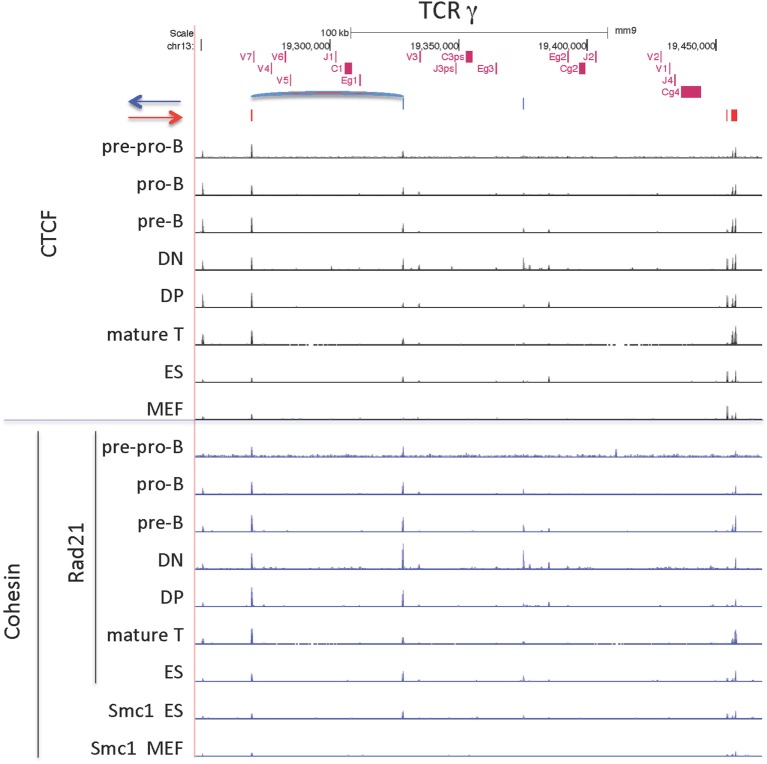
CCCTC-binding factor (CTCF) and cohesin subunit (Rad21 or Smc1) ChIP-seq are plotted for the TCRγ locus, along with the orientation of CTCF sites. Sites of the probable long-range CTCF loop is indicated.

The TCRγ locus is a small locus, encompassing 200 kb, and is composed of three clusters of V-J-C genes, one of which contains only pseudogenes (Figure 10). The main cluster of functional genes contains 4 Vγ genes that are used sequentially during fetal and neonatal life (70). This cluster has one Jγ gene, one Cγ gene, and one enhancer and the entire cluster covers only 41 kb. This main functional cluster is flanked by CTCF sites oriented toward each other that could create a domain *via* a long-range loop between the 2 CTCF sites as indicated in Figure 10. Furthermore, those two CTCF sites have dominant peaks of cohesin as well as CTCF sites bound in all lymphocyte subsets and in ES cells. However, there is some lineage and stage-specificity in that there is low level cohesin binding to this cluster in DN cells, the stage at which this locus rearranges. The TCRγ locus is flanked by relatively conserved CTCF sites, but only the main cluster has convergently oriented sites.

#### Ig Lambda Locus

**Figure 11:**
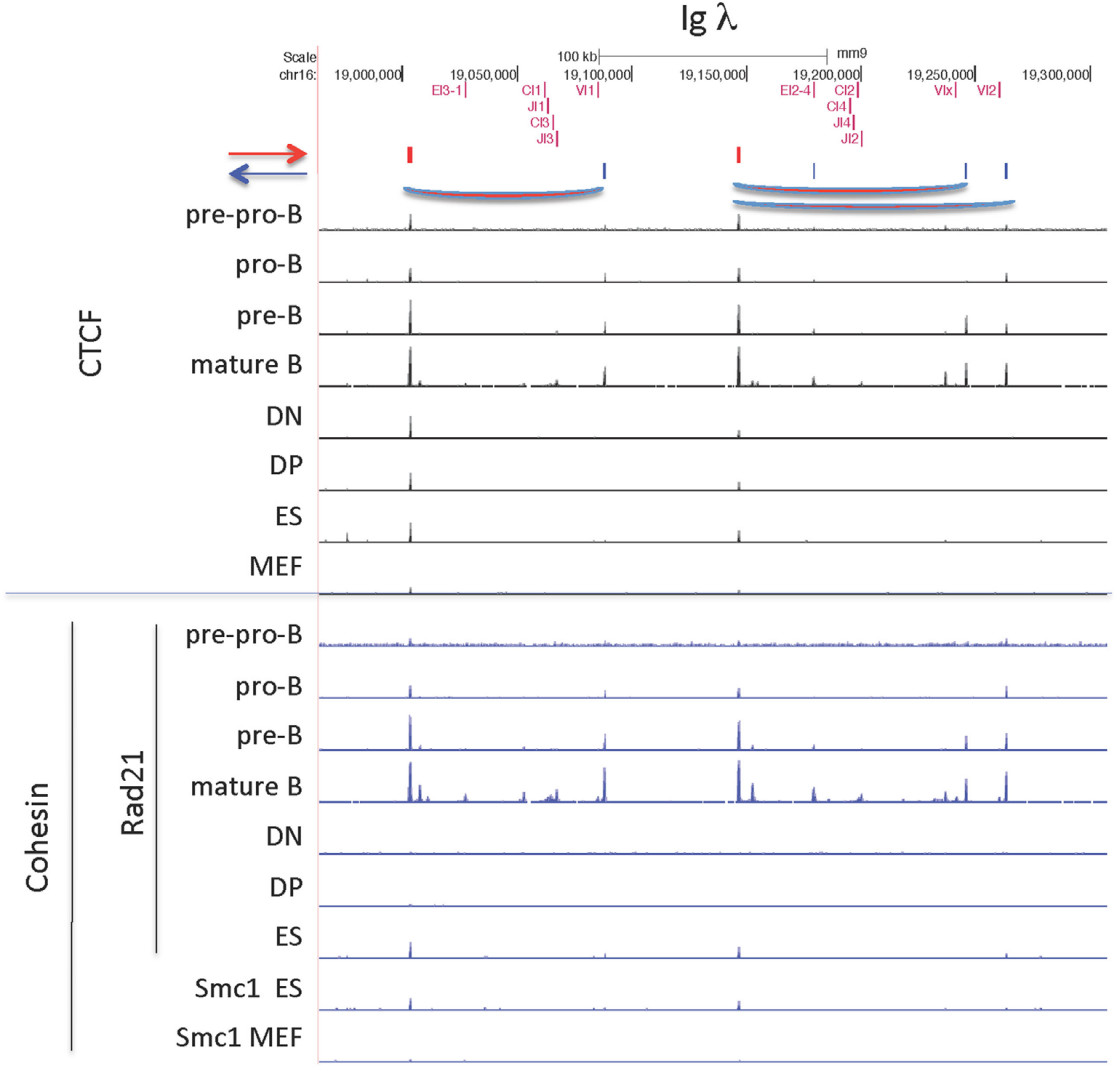
CCCTC-binding factor (CTCF) and cohesin subunit (Rad21 or Smc1) ChIP-seq are plotted for the Igλ locus, along with the orientation of CTCF sites. Sites of probable long-range CTCF loops are indicated.

The Igλ locus, like the TCRγ locus, is organized in clusters, and V genes only rearrange to J genes within their cluster (Figure 11) (71). The first cluster encodes the gene segments encoding the light chains λ1 (Vλ1 + Jλ1 + Cλ1) and λ3 (Vλ1 + Jλ3 + Cλ3), with λ1 being the most commonly used λ gene. This cluster is flanked by convergent CTCF sites, in which the outside downstream CTCF site is universally bound, but the upstream CTCF site flanking Vλ1 is only bound in B lineage cells. Likewise, the other cluster, encoding the λ2 and λx light chains, is also flanked by CTCF sites, but in this case, there is a CTCF site just external to each of the V genes, Vλx and Vλ2, as well as a site downstream of the cluster. As with the first cluster, the site downstream of the enhancer is bound in all cell types, whereas the sites flanking the λ2 gene are bound only in B lineage cells, and the site flanking the Vλx is bound only in pre-B cells, the stage at which lambda rearrangement occurs. Occupancy of all these sites is maintained in mature B cells. The CTCF sites flanking the V genes in this second cluster are each in convergent orientation with respect to the CTCF site downstream of the enhancer. Vλ2 is the second most frequently rearranging Vλ gene, and Vλx is seldom rearranged. This might be because only the CTCF site flanking the Vλ2 gene is bound by CTCF and cohesin as cells enter the pre-B cell stage, creating a loop that would put the Vλ2 gene close to the enhancer end of the cluster. In pro-B cells, the Igh intronic enhancer is in contact with the CTCF sites flanking DFL16.1 and in the 3′ regulatory region (3′RR) (35). If a similar loop is formed here in the second cluster between the flanking CTCF sites and the enhancer that would give preference for rearrangement of the Vλ2 gene rather than the Vλx gene, which is what is actually observed in the repertoire. Cohesin binding in B lineage cells mirrors the CTCF-binding pattern, but there is no cohesin binding in thymocytes, MEFs, or pre-pro-B cells.

## Discussion

The ChIP-seq data presented here show that the CTCF and cohesin bound sites within the four largest AgR loci can be divided into two categories. All four large AgR loci have CTCF sites between V genes and D-J genes, and some have CTCF sites near the enhancers that are usually located downstream of J or C genes and, thus clustered at one end of the locus. With some exceptions, the CTCF sites in these very small D-J-C-enhancer regions of each locus tend to be bound by CTCF in ES cells, and sometimes in MEF, the two non-lymphoid cell types we examined. Some of these CTCF sites have been reported to have insulator function, preventing the spread of active chromatin and keeping germline transcription of proximal V genes low, and deletion of these sites results in preferential rearrangement of proximal V genes (72, 73). Despite having important roles in creating a diverse repertoire of AgR using the full range of functional V genes throughout the AgR locus, these CTCF sites show little lymphocyte specificity in binding. However, not all of these sites have cohesin bound, and thus it is unlikely that these sites without cohesin bound would make effective long-range loops.

By contrast, the majority of the CTCF sites within the large V gene portion of each of those loci, which comprise >85% of the total size of each locus, show lymphocyte specificity in that very few sites are bound in ES cells or MEFs. However, only the Igκ locus shows both lineage (T vs. B cell) and also developmental stage-specificity (pre-B vs. pro-B) for the majority of its bound CTCF sites. This enhanced specificity of CTCF binding may be because, of the four large loci, this is the only one that does not undergo any rearrangement at the first stage of T or B cell differentiation (DN and pro-B, respectively), and thus pre-B cells are characteristic of a later stage of lymphocyte differentiation. ES cells, although having few CTCF sites bound in the AgR loci, often show more binding than the end-stage differentiated MEFs, suggesting that, just as ES cells often have low level transcription of a wide variety of cell type-specific genes, their CTCF binding, although limited, becomes more restricted as ES cells differentiate into non-lymphoid cells. Since DNA methylation has been reported to inhibit CTCF binding, it is possible that the CTCF sites in the AgR loci might be CpG methylated in non-lymphocytes, and only become demethylated in an early lymphoid progenitor stage (74–76). However, the majority of the CTCF sites in the Ig loci do not have CpGs at critical sites within their CTCF motif so this is unlikely to be the reason for the increase in binding with differentiation.

Importantly, we show that Rad21, a component of the cohesin complex, shows greater lineage and developmental stage-specificity in general than CTCF for most of the large AgR loci analyzed for both the V portions of the loci as well as for the several of the CTCF sites in the D-J-C-enhancer regions. Since the loop extrusion model proposes that cohesin is critical to the creation of CTCF-mediated loops, the greater developmental specificity of Rad21 binding to AgR loci suggests substantial developmental specificity in AgR looping (56, 67, 77).

Over the past 3 years, it has become clear that the orientation of the non-palindromic CTCF motif is important in the creation of the long-range loops that mediate most of CTCF’s architectural functions as well as its insulator and enhancer-blocking functions (10, 13, 64, 78). The genome is organized into TADs, and these topological domains are bounded in almost all cases by convergently oriented (facing each other) CTCF sites. The loop extrusion model posits a critical role for cohesin as a loop extruder in the creation of these TAD boundaries (20, 23). Within TADs, subTADs can also be formed which may or may not have CTCF at their boundaries. In many cases, genome-wide CTCF sites are found near enhancers and may help stabilize enhancer–promoter interactions (12). The orientation of CTCF sites forming subTADs is less clear, with more examples of tandem orientation loops (64). The Ig loci, and likely the TCR loci, are present within a single TAD, but the subTAD boundaries are less well defined (43, 66, 79). The Igh locus has at least 2–3 subTADs, and the Igκ locus has a complex 3D chromatin structure as well (43, 66, 79). Within the AgR loci, although it might be hypothesized that the orientation of CTCF sites creating long-range loops may be likely to be convergent, there are already documented exceptions. An ectopic CTCF site was knocked-in in between TCRδ D-J genes and the downstream Trdv5 gene (80). Surprisingly, this ectopic site was oriented toward the convergent TEA CTCF site, but it showed preferential interactions with the tandemly oriented INT2 CTCF sites. Similarly, the CTCF site adjacent to Eα at the far end of the TCRα/δ locus is oriented away from the rest of the locus, yet it interacts with many sites within the TCRα/δ locus (40, 67). Also, in the TCRβ locus, the CTCF site near Eβ has been reported to interact very strongly with the poorly occupied CTCF site just upstream of the promoter of Dβ1, PDβ1, even though those CTCF sites are in tandem orientation. However, the CTCF site near PDβ1 is in the same HindIII fragment as PDβ1, so it is not clear if those 3C experiments are reveal CTCF–CTCF interactions or CTCF interactions with the PDβ1 promoter itself (81). We have previously documented that Eμ is recruited to the interacting CTCF sites at IGCR1 and 3′RR, and the same could be happening here (36). Taken together, it appears that there are several examples of non-convergent CTCF-mediated loops within AgR loci, although it cannot be ruled out that some of these exceptions are mediated in part by enhancers or promoters rather than by CTCF–CTCF loops.

The Igh locus is the subject of the most detailed 3D-FISH analyses. The Igh locus has been demonstrated to be in a rosette-like structure, with three rosettes present in pre–pro-B cells that further contract into one in pro-B cells, the stage at which rearrangement of that locus takes place (28). It is likely that the other AgR loci also are composed of many long-range loops, and it is known that all the large AgR loci adopt a more contracted structure before they undergo rearrangement, although not precisely at the initiation time of rearrangement, and in some cases, not throughout the entire locus (30, 31, 33, 34, 82). We and others had proposed that the many CTCF sites within the Igh locus could create this rosette structure (35, 36, 83). We now know that all of the bound CTCF sites within the 2.5 Mb Vh region portion of the locus are oriented toward the D-J-C regions, and at the far 3′ end of the locus are a cluster of nine CTCF sites known as the 3′ regulatory region (3′RR), all of which point toward the V regions (Figure 8) (66, 84). The only other CTCF sites in the Igh locus are a pair of CTCF sites located ~3.1 and 5.7 kb 5′ of the most J-distal functional D gene, DFL16.1 (35). One of these sites is oriented toward the V genes, while the other one is oriented toward the 3′RR (72, 84). We had proposed several years ago that a loop could be made between these CTCF sites adjacent to DFL16 with those of the 3′RR to create a looped domain containing all of the D and J gene segments but excluding all of the V genes (35). We further proposed that this could allow D to J joining without V to DJ joining, and thus would facilitate ordered rearrangement. Our 3C studies and those of others confirmed the existence of this CTCF loop (36, 72). Since all of the bound CTCF sites within the 2.8 Mb V region are oriented toward the nine sites at the 3′RR, it has more recently been proposed that the rosette structure at the Igh locus is formed by CTCF sites throughout the Vh portion of the locus each directly interacting with the 3′RR, which has been termed a superanchor (66). Therefore, in this model, there are no CTCF-mediated loops between pairs of CTCF sites within the 2.8 Mb V region portion of the Igh locus itself. However, 4C analysis by Medvedovic et al. indicated that there is a continuum of loops across the entire Igh locus, while the 5C studies of Montefiori et al. give defined boundaries to the sub-domains of the Igh locus (60, 79). Some of these loops could be created by elements other than CTCF, but to the extent that the loops in either study are CTCF-mediated, the role of convergent vs. tandem CTCF loops remains unclear for the Igh locus.

The much smaller TCRβ locus, such as the Igh locus, has all of the CTCF sites within the V region part of the locus oriented toward the D-J gene segments (Figure 9). There are three CTCF sites in the D-J portion of the locus, all facing the many CTCF sites in the V portion. The tallest peak of the three (called 5′PC) is 27 kb upstream of Dβ1.1, and was described as being essential for long-range interactions of the D genes with distal V genes and for rearrangements of those distal V genes (56). A much lower CTCF peak is located ~3 kb upstream of Dβ1.1, and its deletion results in the spread of the active chromatin marks up to 5′PC, and also prevents 5′PC from making long-range interactions with distal Vβ genes (56). However, in wild-type DN cells, the active histone marks extend ~6 kb upstream of Dβ1.1, stopping at an long-terminal repeat (LTR) element which appears to be the barrier element (85), so the relationship of the LTR with the weak CTCF site 3 kb downstream of the LTR for the barrier function is not clear. Nonetheless, it is clear that these two CTCF sites, but not the Eβ enhancer, are required for the long-range looping of distal half of the V locus to the J regions.

Igκ locus has many CTCF sites within the 3.1 Mb V region portion of the locus oriented in both directions, although most of the sites in the proximal half of the locus are oriented away from the CTCF sites in the V-J intervening region, and most of those are weakly if at all bound in pro-B cells, although they are bound in IL7-cultured pro-B cells. The orientation pattern would predict that the CTCF sites at Cer would predominantly interact with sites in the distal half of the locus if convergent orientation were important, and we know that Cer is essential for locus contraction (55). Since the Igκ locus is already contracted, as measured by 3D-FISH, even in non-IL7-cultured pro-B cells, the CTCF sites that are bound in pro-B cells in the distal half of the locus that are also oriented toward the CTCF sites in Cer may well be critical for creating long-range loops facilitating locus contraction (32). Hi–C analysis of IL7-cultured RAG^−/−^ pro-B cells shows many interactions within the Igκ locus, but there is a paucity of interactions from Cer/Sis/iEκ region to a large portion of the proximal half of the locus, from ~Vκ12-46 to Vκ4-83. Although CTCF sites throughout the locus are bound in IL7-cultured pro-B cells, this portion of the locus with the fewest Hi–C interactions within the proximal part of the locus lack any CTCF sites oriented toward Cer/Sis (43). However, the Hi–C does show interactions of Cer/Sis/iEκ region with the region containing the first 41 Vκ genes, and there are bound CTCF sites oriented in the proper convergent orientation in that region. By contrast, 4C analysis of fresh *ex vivo* RAG^−/−^ pro-B cells show many fewer sites of interaction with Sis, iEκ, and 3′Eκ at the 3′ half of the locus despite the presence of some CTCF sites oriented toward Cer, consistent with the minimal binding of CTCF to the proximal half of the locus in *ex vivo* pro-B cells (77). Our data indicating that IL7 culture of pro-B cells results in increased binding of CTCF and cohesin to the proximal half of the locus, and that it induces epigenetic and transcriptional characteristics of pre-B cells (Figure 4), may partially explain that discrepancy.

Long-range looping interactions are not all mediated by CTCF and cohesin. It is well established that enhancers and promoters make looping interactions, and indeed gene activation and transcription are critical in forming chromosomal compartments, structures which are not dependent upon CTCF (46, 86–88). AgR loci undergo robust non-coding RNA transcription, called “germline transcription,” through the J and constant regions at the particular stage of differentiation when that locus is undergoing V(D)J recombination (89). In addition, there is both sense and antisense, genic and intergenic, non-coding transcription happening, usually at low levels, throughout the large V region portions of each AgR locus (61, 90, 91). Thus, it is possible that germline transcription can result in a change in the topology of the AgR loci since multiple transcribing regions within an AgR loci may well be located in a transcription factory where other regions of the AgR loci, especially the J-C regions which undergo robust germline transcription, may also be located (61, 92). Also, non-coding RNA has been shown to be capable of making large scale alterations in chromatin structure by allowing the recruitment of CTCF to sites which otherwise did not have it bound, creating long-range CTCF-mediated interactions (93). Thus, CTCF and its partner cohesin are not the only factors that are competent to produce long-range looping and to contribute to 3D chromatin conformation in the AgR loci.

In order to create a diverse repertoire of antibodies or TCRs, the AgR loci become more compact at the time of rearrangement to bring the many V genes, spread over large genomic space, into contact with the (D)J genes to which one V gene will rearrange. We have previously shown that CTCF is important for the contracted structure found at the Igh locus in pro-B cells, since knock-down of CTCF shows a more extended Igh locus (36). However, it is known that the transcription factors Pax5 and YY1 are essential for making long-range loops essential for locus contraction (79, 94) and we have data for the influence of novel enhancer elements in creating long-range loops within the Igκ locus (EB-M and AF, unpublished data). Furthermore, once CTCF and cohesin become bound at any of the AgR loci, we show here that the pattern of binding remains stable from pre-B to mature B or from DP thymocytes to mature T cells even though at least for the Igh and TCRβ loci, it has been demonstrated that those loci de-contract at the next stage of differentiation. Hence, we hypothesize that the onset of lymphocyte-specific CTCF/cohesin binding throughout the V region portion of the AgR loci in lymphocytes can set up an early level of 3D structure for a locus. However, it is likely that transcription factors, possibly bound to novel or traditional enhancers, will ultimately determine the presence or absence of a contracted AgR locus, since it is clearly not the case that the CTCF/cohesin binding pattern reverts to that of pre–pro-B cells or of non-lineage-specific binding after each locus has finished V(D)J rearrangement.

In order to efficiently utilize the large number of V genes in each loci, it seems reasonable that there should be heterogeneity in the long-range looping interactions that bring the V genes close to the (D)J gene such that different V genes are closest to the (D)J gene in the population of precursor lymphocytes undergoing V(D)J recombination. This need for heterogeneity could suggest that long-range loops are constantly forming and reassembling in each precursor lymphocyte to create a structure in which different V genes are closest to the (D)J genes at different times, and there is evidence that CTCF binding, and by inference chromatin looping, is very dynamic (24). Alternatively, it could be that there is extensive heterogeneity within the population of lymphocyte precursors of long-range loops that might be somewhat more stable, as was suggested in studies on long-term cultured pre-B cells (95). The fact that the height of most of the bound CTCF peaks in the AgR loci are low might suggest that only a subset of the millions of cells analyzed in a ChIP-seq have CTCF bound at those individual sites at any given time, possibly due to weaker affinity for specific CTCF sites. If the majority of CTCF sites within the AgR loci had lower affinity, this could serve an important function in allowing the random loose creation and dissolution of many CTCF-mediated loops, providing the opportunity for a dynamic and changing heterogeneous set of loops in each precursor lymphocyte, which would help facilitate the creation of diverse immune B and T cell repertoires, utilizing V genes throughout the large AgR loci.

## Ethics Statement

This study was carried out under approval of our protocol by The Scripps Research Institute’s IACUC.

## Author Contributions

SL performed all of the bioinformatic analyses. SL, EB-M, and AF analyzed all of the data. EB-M performed some of the ChIP. H-YS and MK provided the data in Figure S2B in Supplementary Material. AF and EB-M prepared the figures. AF, EB-M, and MK wrote the manuscript with input from all the authors, especially SL.

## Conflict of Interest Statement

The authors declare that the research was conducted in the absence of any commercial or financial relationships that could be construed as a potential conflict of interest.

## Abbreviations

AgR loci, antigen receptor loci; Ig, immunoglobulin; BM, bone marrow; TAD, topologically associating domains.
