# Supplementary material for: Variable Extent of Lineage-Specificity and Developmental Stage-Specificity of Cohesin and CCCTC-Binding Factor Binding Within the Immunoglobulin and T Cell Receptor Loci
Source: Front Immunol. 2018 Mar 8;9:425. doi: 10.3389/fimmu.2018.00425 (PMC5859386; doi:10.3389/fimmu.2018.00425)
Supplement: Supplementary file 1 [file Presentation_1.PDF]

Fig. S1a

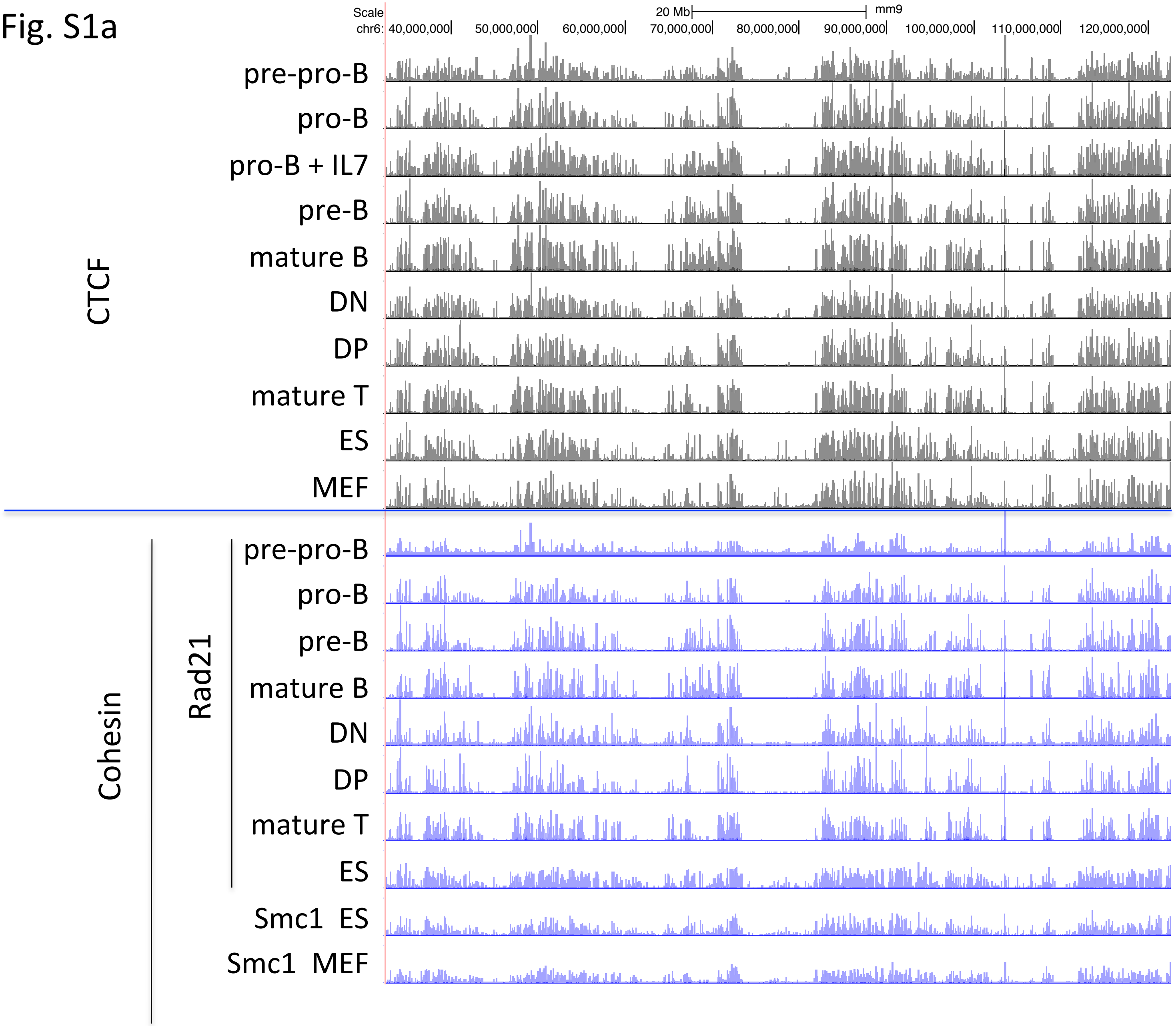

Fig. S1 Genome Browser views of all analyzed ChIP-seqs in 90 kb windows in (A) chr 6 and (B) chr 12.

Fig. S1b

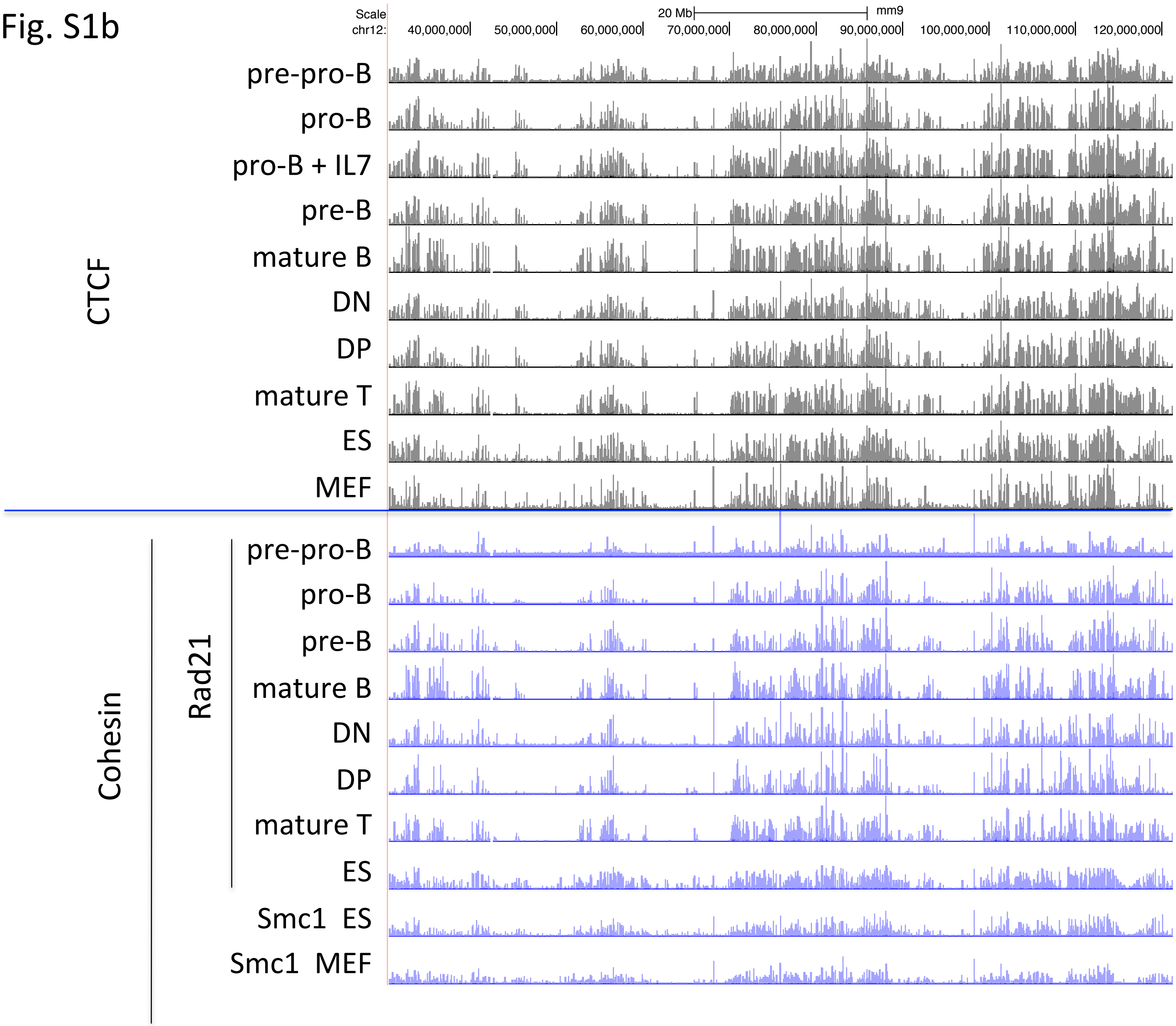
Scale
20 Mb
mm9
chr12:
40,000,000
50,000,000
60,000,000
70,000,000
80,000,000
90,000,000
100,000,000
110,000,000
120,000,000
CTCF
pre-pro-B
pro-B
pro-B + IL7
pre-B
mature B
DN
DP
mature T
ES
MEF
Cohesin
Rad21
pre-pro-B
pro-B
pre-B
mature B
DN
DP
mature T
ES
Smc1 ES
Smc1 MEF

Fig. S2

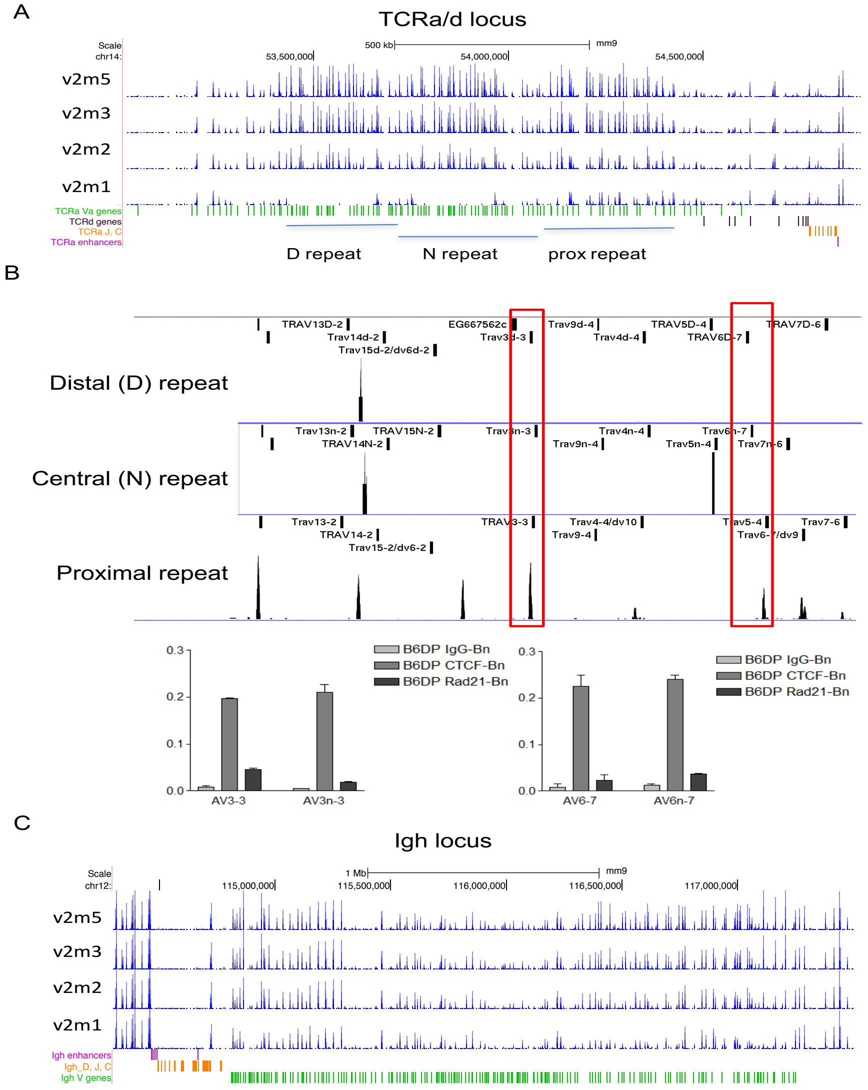

Fig. S2 Parameters for mapping ChIP-seq reads at the TCRα/δ (A) and Igh (C) loci. The same CTCF ChIP-seq was analyzed with the parameters v2m1 (only keeping uniquely mapping reads), v2m2 (keeping reads if bound to no more than 2 locations within the AgR locus), v2m3 and v2m5. (A) CTCF ChIP-seq was done on DP thymocytes. At the TCRα/δ locus, it can be seen that v2m1 parameter greatly eliminates binding of sites in the distal 2/3 of the locus containing the d and n duplications, and even v2m2 shows less binding than v2m3. The v2m5 parameter is similar to v2m3 parameter. (B) ChIP/qPCR for assessing binding at the triplicated region of the TCRα/δ locus with primers distinguishing the d/n repeats from the 3' copy demonstrates equivalent binding at these regions. (C) CTCF ChIP-seq was done on pro-B cells. Although the pattern of reads is similar with all 4 parameters, the total read count is slightly lower at the distal portion of the locus containing the large VhJ558 family.
