# Supplementary material for: Variable Extent of Lineage-Specificity and Developmental Stage-Specificity of Cohesin and CCCTC-Binding Factor Binding Within the Immunoglobulin and T Cell Receptor Loci
Source: Front Immunol. 2018 Mar 8;9:425. doi: 10.3389/fimmu.2018.00425 (PMC5859386; doi:10.3389/fimmu.2018.00425)
Supplement: Supplementary file 4 [file Table_3.docx]

**Supplemental Table 3 CTCF motifs**

Library of CTCF PWMs include:

- Five PWMs derived to accommodate the divergence of CTCFBS sequences, included in the InsulatorDB CTCFBS Prediction Tool (<http://insulatordb.uthsc.edu/storm_new.php>);

See also <http://insulatordb.uthsc.edu/help_new.php>.

- Four PWMs obtained after running HOMER motif detection on both pre-B and pro-B confident ChIP-seq peaks (found three known motifs + one detected de novo).

| Motif | Name | Reference |
| --- | --- | --- |
| CGCCCCCTGGTGGC | EMBL_M1 | ([1](#_ENREF_1)) |
| GTGGCCAGGAGAGGGAGCCA | REN_20 | ([2](#_ENREF_2)) |
| TTAACACAAGATGGCAGCA | MIT_LM2 | ([3](#_ENREF_3)) |
| CAGCCAGGAGGTGGGGCTGT | MIT_LM7 | ([3](#_ENREF_3)) |
| CCGCCACTAGATGGTGCTAA | MIT_LM23 | ([3](#_ENREF_3)) |

| Motif | Name | Reference | Details |
| --- | --- | --- | --- |
| AYAGTGCCMYCTRGTGGCCA | Homer_Known1 | ([4](#_ENREF_4)) | CTCF(Zf)/CD4+-CTCF-ChIP-Seq |
| CNNBRGCGCCCCCTGSTGGC | Homer_Known2 | GSM803401 | BORIS(Zf)/K562-CTCFL-ChIP-Seq |
| TGCAGTTCCMVNWRTGGCCA | Homer_Known3 | ([4](#_ENREF_4)) | CTCF-SatelliteElement(Zf)/CD4+-CTCF-ChIP-Seq |
| GCCCCCTRGTGG | Homer_DeNovo | GSM803401 | BestGuess:BORIS(Zf)/K562-CTCFL-ChIP-Seq |

1. Schmidt, D., P. C. Schwalie, M. D. Wilson, B. Ballester, A. Goncalves, C. Kutter, G. D. Brown, A. Marshall, P. Flicek, and D. T. Odom. 2012. Waves of retrotransposon expansion remodel genome organization and CTCF binding in multiple mammalian lineages. *Cell* 148:335-348.

2. Kim, T. H., Z. K. Abdullaev, A. D. Smith, K. A. Ching, D. I. Loukinov, R. D. Green, M. Q. Zhang, V. V. Lobanenkov, and B. Ren. 2007. Analysis of the vertebrate insulator protein CTCF-binding sites in the human genome. *Cell* 128:1231-1245.

3. Xie, X., T. S. Mikkelsen, A. Gnirke, K. Lindblad-Toh, M. Kellis, and E. S. Lander. 2007. Systematic discovery of regulatory motifs in conserved regions of the human genome, including thousands of CTCF insulator sites. *Proc Natl Acad Sci U S A* 104:7145-7150.

4. Barski, A., S. Cuddapah, K. Cui, T. Y. Roh, D. E. Schones, Z. Wang, G. Wei, I. Chepelev, and K. Zhao. 2007. High-resolution profiling of histone methylations in the human genome. *Cell* 129:823-837.
